# Use of cluster-graphs from spoligotyping data to study genotype similarities and a comparison of three indices to quantify recent tuberculosis transmission among culture positive cases in French Guiana during a eight year period

**DOI:** 10.1186/1471-2334-8-46

**Published:** 2008-04-14

**Authors:** Vanina Guernier, Christophe Sola, Karine Brudey, Jean-François Guégan, Nalin Rastogi

**Affiliations:** 1 UMR 2724 IRD-CNRS, Génétique et Évolution des Maladies Infectieuses, Equipe Dynamique des Systèmes & Maladies Infectieuses, 911 avenue Agropolis, BP 64501, 34394 Montpellier Cedex 05, France; 2 Unité de la Tuberculose et des Mycobactéries, Institut Pasteur de Guadeloupe, BP 484, 97183 Abymes Cedex, Guadeloupe; 3 Institut de Génétique et de Microbiologie UMR8621, CNRS, Université Paris-Sud 11, 91405 Orsay, France; 4 Unité de Génétique Mycobactérienne, Institut Pasteur, 25 rue du Dr Roux, 75724 Paris Cedex 15, France; 5 Centre Hospitalier Universitaire, Route de Chauvel, BP 465, 97139 Pointe-à-Pitre Cedex, Guadeloupe

## Abstract

**Background:**

French Guiana has the highest tuberculosis (TB) burden among all French departments, with a strong increase in the TB incidence over the last few years. It is now uncertain how best to explain this incidence. The objective of this study was to compare three different methods evaluating the extent of recent TB transmission in French Guiana.

**Methods:**

We conducted a population-based molecular epidemiology study of tuberculosis in French Guiana based on culture-positive TB strains (1996 to 2003, n = 344) to define molecular relatedness between isolates, *i.e. *potential transmission events. Phylogenetic relationships were inferred by comparing two methods: a "cluster-graph" method based on spoligotyping results, and a minimum spanning tree method based on both spoligotyping and variable number of tandem DNA repeats (VNTR). Furthermore, three indices attempting to reflect the extent of recent TB transmission (RTI*n*, RTI*n-1 *and TMI) were compared.

**Results:**

Molecular analyses showed a total amount of 120 different spoligotyping patterns and 273 clinical isolates (79.4%) that were grouped in 49 clusters. The comparison of spoligotypes from French Guiana with an international spoligotype database (SpolDB4) showed that the majority of isolates belonged to major clades of *M. tuberculosis *(Haarlem, 22.6%; Latin American-Mediterranean, 23.3%; and T, 32.6%). Indices designed to quantify transmission of tuberculosis gave the following values: RTI*n *= 0.794, RTI*n-1 *= 0.651, and TMI = 0.146.

**Conclusion:**

Our data showed a high number of *Mycobacterium tuberculosis *clusters, suggesting a high level of recent TB transmission, nonetheless an estimation of transmission rate taking into account cluster size and mutation rate of genetic markers showed a low ongoing transmission rate (14.6%). Our results indicate an endemic mode of TB transmission in French Guiana, with both resurgence of old spatially restricted genotypes, and a significant importation of new TB genotypes by migration of TB infected persons from neighgouring high-incidence countries.

## Background

The resurgence of human tuberculosis (TB) in many industrialized countries in the late 1980s and early 1990s [1] has focused critical attention on a disease that was thought to be under control, and as such, has been neglected for too long. Effective prevention and control of *Mycobacterium tuberculosis*, the causative agent of TB, must be based on a clear understanding of how the disease is transmitted, how infection becomes established, and how infection progresses to clinical disease. This has motivated investigations on TB transmission among persons at higher risk, such as the homeless [2,3] or HIV-infected persons [4,5]. Furthermore, the improvement of molecular techniques to characterize *Mycobacterium *strains, especially *M. tuberculosis*, and the concomitant accumulation of epidemiological data over large spatial scales has increased the possibility to trace transmission routes of *Mycobacterium *outbreaks (e.g. *M. leprae *in [6]). The identification of *M. tuberculosis *strains can be used to identify patients which have some apparently identical genotypes, thus potentially associated to a same source of contamination [7] and designated as «clustered cases» [8,9]. The existence of genetic clusters is thought to be indicative of recent TB transmission [10,11], in opposition to cases arising either from imports or from reactivation of latent infection. Featuring epidemiological links between patients allow inferences about the level of ongoing TB transmission, thus the level of epidemicity/endemicity of the infection in a defined geographical area, and underline some potential risk factors associated to the disease [12]. For example, when an unusual number of TB cases occurs over time, it is possible to do early molecular analyses to determine whether this cluster of cases represents temporal coincidence, or a genetic cluster due to a local chain of transmission. If fingerprinting demonstrates different strains, the cases are not due to transmission, and there is no need for further epidemiological evaluation.

The underlined hypothesis of the use of molecular information for the evaluation of ongoing transmission is that mutations are slower than transmission rate [13], which is not true for all genetic markers. A large number of molecular methods are available for the characterization of infra-specific *M. tuberculosis *strains. Nonetheless the choice of a method strongly affects the results, as low mutation rates tend to less discriminate the genetic differences, whereas high mutation rates tend to increase the number of clusters considered [14]. This article investigates the genotypic diversity of *M. tuberculosis *strains isolated in French Guiana over the 1996–2003 period, characterized by two molecular methods, and attempts to determine potential transmission between infected persons. We used a recent method proposed by Tanaka & Francis [14] to visualise molecular epidemiological data, *i.e. *cluster-graphs, which also provides a framework for evaluating ongoing transmission. We also compared the method used to previous indices attempting to reflect the extent of recent TB transmission.

## Methods

### Clinical isolates

French Guiana is a French overseas department of 32,432 sq mi. It is located on the north-east of South America, between Suriname and Brazil. The region has the highest TB rates among all French departments, with an average annual incidence of 63.1 TB cases per 100,000 population estimated during the period 1996–2003 [15]. Among all TB cases identified from January 1, 1996 to December 31, 2003, 345 were confirmed by isolation and cell culture of *M. tuberculosis *by the Mycobacterium Laboratory of the Institut Pasteur de la Guyane (IPG). A total of 344 clinical isolates of *M. tuberculosis *were isolated from patients with culture-positive TB, representing 342 patients (two patients only were contaminated twice, by two different strains). The three missing cases (0,86%) correspond to contaminated cultures. After a prior explanation of the recommended investigations for the diagnostic purposes by a physician, patients were asked to provide oral consent and the pathological specimens were obtained from suspected tuberculosis patients residing in French Guiana. The cultures were performed using Löwenstein-Jensen slants at 37°C at the Pasteur Institute of French Guiana. Positive cultures (where no identification was attached) were sent to the Pasteur Institute of Guadeloupe which has been serving as a regional reference laboratory for tuberculosis and mycobacterial for the Caribbean since 1994. The cultures were duly identified as *M. tuberculosis *complex using classical biochemical tests and the AccuProbe test (GenProbe Inc., San Diego, CA) and subjected to drug-susceptibility testing using the proportional method. The bacteriological results were communicated to the physicians and/or hospital services responsible for patient care and treatment, through the Pasteur Institute of French Guiana. DNA-based molecular typing was also performed (see below).

### Molecular typing

Molecular typing was performed using spoligotyping and VNTRs analyses. It was previously suggested that a combined spoligotyping-5 VNTR loci genotyping scheme may have a discriminatory power close to that of IS6110 RFLP, the historical "golden standard" in tuberculosis genotyping [16]. Hence, for ease and resources reasons, we choosed this scheme, which was less expensive in time and money than the combined spoligotyping-12 VNTR loci scheme, *i.e. *MIRUs [17]. Recent improvement in VNTR typing now recommended a new "universal" 15 or even 24 VNTR loci genotyping scheme for *M. tuberculosis *[18].

The bacterial DNA was prepared by the classical cetyl-trimethyl ammonium bromide method (CTAB) [19] and used for spoligotyping (spacer oligonucleotide typing) and Variable Number of DNA Tandem Repeats (VNTRs) analyses. Spoligotyping was performed using a home-made membrane with 43 covalently bound oligonucleotides and PCR was achieved using primers designated DRa and DRb, with DRa biotinylated 5' to amplify the whole DR region as described previously [20]. As spoligotyping used alone is known to overestimate the number of epidemiological links (depending on the settings, but by around 30%), it was suggested to be used in association with another rapid fingerprinting technique [21]. Hence, the isolates clustered by spoligotyping (and only those first-clustered isolates) were further subtyped using VNTRs as described previously [22] in order to provide a second independent indicator of clonality. The number of copies for each exact tandem repeat (ETR) was documented as a five-digit numbers representing allele profiles ETR-A to ETR-E [23].

### Strains matching

Spoligotype patterns are designated as 43-character-long strings consisting of black and white squares, indicating respectively the presence or the absence of an individual spacer. The totality of the 344 isolates was entered in this binary format as Excel (Microsoft, Cupertino, CA) spreadsheets. The spoligotype designations (shared types or ST, defined as an identical spoligotype found in ≥ 2 individual patient isolates) were attributed by comparing the patterns obtained with those included in an international spoligotyping database held at the Pasteur Institute of Guadeloupe, designated as spolDB4 [24]. At the time of the matching analysis, the updated spolDB4 version contained 31,642 patterns distributed into 2,393 shared types in 114 countries (an online version of this database is now available at ). Patterns referenced only once are designated as true orphans (in opposition to pseudo-orphans, *i.e. *isolates found to be unique in this study but for whom a counterpart exists in the database) and are not labelled with an ST number. Major phylogenetic clades were assigned to STs according to signatures provided in SpolDB4 (the reader is referred to the original paper [24] for a detailed description). These included specific signatures for various *M. tuberculosis *sub-species (*M. bovis*, *M. microti, M. caprae, M. pinipedii*, *M. africanum*), as well as rules defining major lineages/sub-lineages for *M. tuberculosis *stricto sensu. The latter included the Central-Asian (CAS) clade and 2 sub-lineages; the East-African-Indian (EAI) clade and 9 sub-lineages; the Haarlem (H) clade and 3 sub-lineages; the Latin-American-Mediterranean (LAM) clade and 12 presumed sub-lineages; the "Manu" family and 3 sub-lineages; the S clade; the IS*6110*-low banding X clade and 3 sub-lineages; and an ill-defined T clade with 5 sub-lineages. The T clade corresponds to Principal Genetic Groups (PGG) 2 and 3, and remains defined by default (no specific signature sequences). *M. tuberculosis *families are phylogeographically-specific, as generally indicated by their names.

### Phylogeography and cluster-graphs

Data were analysed by grouping isolates into clusters of identical genotypes and by organizing all genotypes present in the overall sample according to genetic relationships, *i.e. *by constructing a phylogeny. We used and compared two methods for inferring potential phylogenetic relationships: (1) a classification based on spoligotyping results, which we call a "cluster-graph" and which is based on parsimony principles, and (2) a minimum spanning tree (MST) method which is based on a spoligotyping and VNTR combined distance matrix, computed using the BioNumerics software (Applied Maths, Sint Marten-Latems, Belgium).

**Figure 1:**
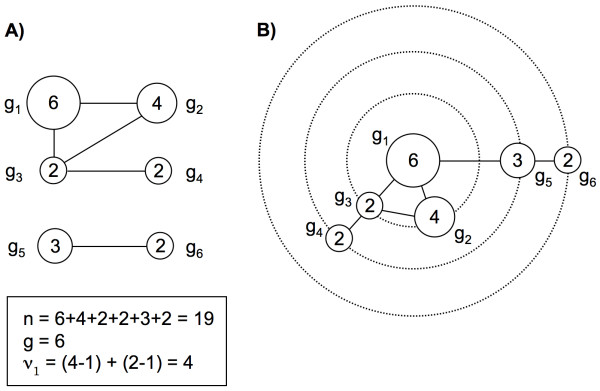
**Cluster-graph representing a sample of 19 TB cases with six distinct genotypes**. Each node represents a cluster of genotypically identical isolates, and the sizes of clusters are indicated inside the nodes. (A) Illustration from Tanaka and Francis (2005). The arrow indicates the sense of evolution through mutation events. (B) Our own representation. Dotted circles indicates mutation steps, with one mutation step between g1 and g3, but two between g1 and g4, for instance.

A cluster-graph is a new graphic illustration developed by Tanaka and Francis [14] for representing and analysing clonal pathogenic genotypes. The graphic tree is drawn with genotypes at internal nodes, edges reflecting direct evolutionary relationships between them, and the size of the circle at the nodes being proportional to cluster sizes. The first step is to form clusters of *g *distinct genotypes (*g *nodes), then to connect all vertices whose genotypes are separated by a single mutation step [14]. In our study, connections between genotypes separated by more than one mutation step were visualized within concentric successive circles which illustrated the increased number of mutation events gained from inside to outside. The Figure 1 illustrates the difference between cluster-graph published by Tanaka and Francis, and the adaptation that we made in the present exercise. The "BioLayout" software was used to draw the networks (cluster-graph) representing genotype similarities, similarly as what can be done for proteins considering their amino-acid content [25,26].

The cluster-graph method could not be applied to the combined spoligotyping-VNTR data, as the manual inference of tree edges between genotypes needs to be supported by the number of mutations events between different genotypes. The VNTR analyses giving occurrence of ETR-A to E do not give any information about the genetic distance between different VNTR patterns. VNTR analyses were included on the construction of the minimum spanning tree method, in order to provide further discrimination between strains for isolates with identical spoligotyping results, Distance matrices are calculated and the obtained groupings can be used to build a dendrogram.

### Estimation of transmission

Three indices of outbreak severity were calculated and compared. The two first ones are referred to as recent transmission indices (RTI) because they are intended to reflect the extent of recent transmission of tuberculosis [14]. The first index is RTI_*n *_= *n*_*c*_/*n *[10], where *n *is the total number of cases in the sample and *n*_*c *_is the total number of cases in cluster (size two or greater). The second one is RTI_*n*-1 _= (*n*_*c *_- *c*)/*n *[11], where *c *is the number of genotypes represented by at least two cases; *i.e. *one case per cluster, which is supposed to be due to reactivation of a previous TB case, is taken off. These have been referred to as the "n method" and the "n-1 method" respectively [27,28]. The underlined idea is that a more genetically homogeneous data set would represent a more severe extent of disease transmission, *i.e. *an "outbreak". The third index is the transmission mutation index (TMI) [14].

$$TMI=\tilde{\mu }\frac{\left(n-g+\nu_{1}\right)}{\nu_{1}}$$

where $\tilde{\mu }$ is an independent estimate of the mutation rate of the genetic marker, and *v*_1 _is the number of single-step mutation events inferred from the data [14]. We used a mutation rate for spoligotypes of 0.039 events per year computed from previous estimates in the literature [14,29]. All three indices are expressed as a ratio from 0 to 1, which can also be expressed in percentage. It must be interpreted in terms of "percent of isolates in cluster", which is indicative of the rate of recent TB transmission. Thus, a result of 0.5 indicates that 50% of the isolates of a data set were probably related to a recent transmission event.

## Results

### Molecular typing results

The spoligotyping was performed as a first-line screening method on 344 *M. tuberculosis *clinical isolates from patients living in French Guiana, followed by VNTR performed on 273 clustered isolates. Among the 344 typed isolates, spoligotyping generated 49 clustered patterns totalling 273 isolates (with 2 to 48 patients per cluster), 25 true orphan patterns, and 46 pseudo-orphans, *i.e. *patterns already found in SpolDB4 but present as single in this study, with a total amount of 120 different patterns. Analysis of the frequency of major spoligotypes with SpolDB4 allowed a differentiation between ubiquitous types present in all the continents and types more specific to French Guiana. Several types were endemic in French Guiana (*i.e. *ST 66, 76, 94, 1084, 1340, 1526 and 25 orphan isolates), representing 25.8% of the shared-types, and 12.2% of the total isolates. Ubiquitous types represent 40.8% of the 120 individual patterns, *i.e. *70.6% of the isolates.

**Figure 2:**
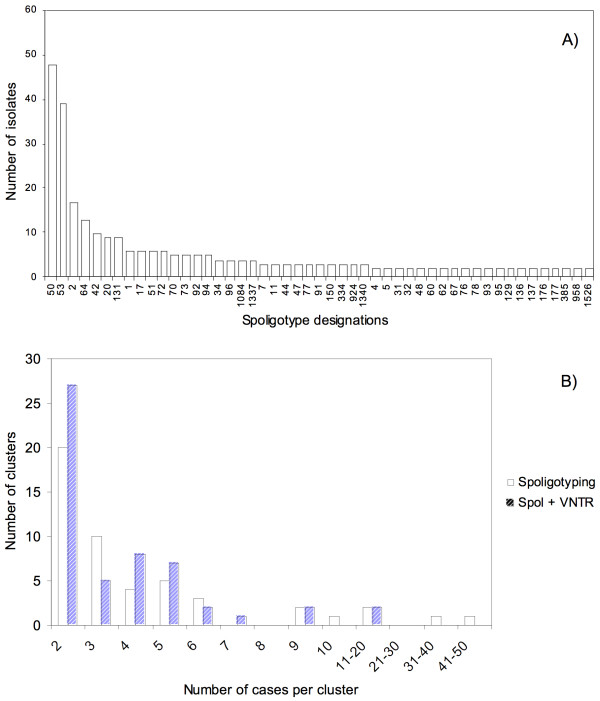
**Synthetic histograms of the distribution of all isolates**. (A) Frequency distribution of isolates associated to the 49 spoligotypes (STs) censused at least two times in French Guiana, southern America, over the 1996–2003 period; the most represented spoligotype is ST 50, with 48 isolates. (B) Frequency distributions of clusters ranked by cluster sizes, when clustered by spoligotyping technique (opened histograms), or when considering combined results of spoligotyping and VNTR (dashed histograms) (derived from table 1).

Among the 49 clusters, we found 34 minor spoligotypes (including two to four isolates), and 15 major spoligotypes (five or more isolates). ST 66, 72, 385, 958, 1337, 1339, 1340, 1486, 1762, 1763, and 1935 are over-represented in French Guiana, *i.e. *they represents more than 50% of total number of isolates associated to these STs within the international spoligotype database SpolDB4. When spoligotypes were subtyped by VNTRs-excluding 14 isolates for which at least one VNTR failed – the number of cases per cluster decreased whereas the number of clusters slightly increased (52 clusters). Moreover, 17 spoligotype clusters on 49 were non-polymorphic for VNTR, *i.e. *all cases of a same spoligotype cluster shared the same VNTR profile. The main results of the molecular typing analysis are summarized on Table 1 and Figure 2. If we examine VNTR results of the five largest spoligotype clusters (including ten to forty-eight isolates), they were subdivided in 21 clusters (including two to twenty-four isolates) and 14 individual patterns (see Table 2 for details).

**Table 1:** Number and frequencies of isolates clustered by spoligotyping alone and further subtyped by VNTRs.

	**Isolates clustered by spoligotyping**	**Isolates clustered by spoligotyping and VNTR**
Total number of isolates studied	344	339
Number of clusters	49	52
Mean number of isolates per cluster	5.57	3.90
Number of clustered isolates (and %)	273 (79.4%)	203 (59.9%)
Number of unclustered isolates (and %)	71 (20.6%)	127 (37.5%)

**Table 2:** Detailed results of clustering for the five largest spoligotype clusters with combined spoligotyping – 5 VNTR loci genotyping.

**Spoligotype**	**N**	**Number of different VNTR**	**Number of isolates/cluster**	**Individual patterns**
ST 53	39	14	6, 5, 5, 4, 4, 3, 2, 2, 2, 2	4
ST 50	42	8	24, 7, 4, 2, 2	3
ST 42	10	7	3, 2	5
ST 64	13	4	9, 2	2
ST 02	15	2	11,4	0

### Phylogeographic analysis

**Figure 3:**
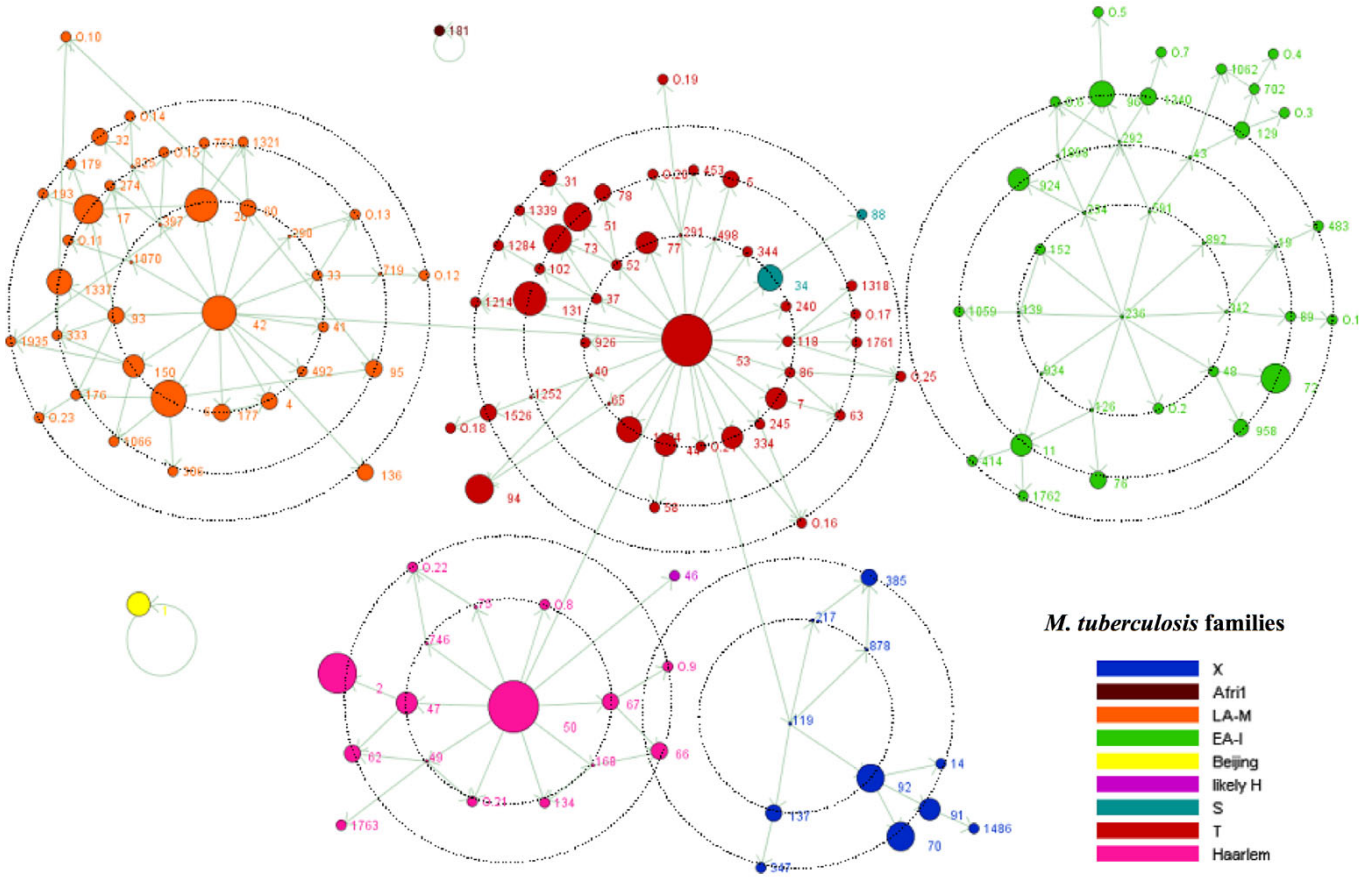
**Cluster-graph drawn from spoligotyping data of 344 TB cases censused in French Guiana over the 1996–2003 period**. Each node represents a genotype, and arrows point in the direction of genotypes possibly derived by deletion in the marker locus. Vertex (or node) size indicates the size of the cluster. Twenty nine non-censused spoligotypes, which are hypothetical intermediates or ancestors of censused spoligotypes, were added and schematised as a point (*e.g. *STs 119 and 236, putative ancestors of X clade and EAI clade, respectively).

**Figure 4:**
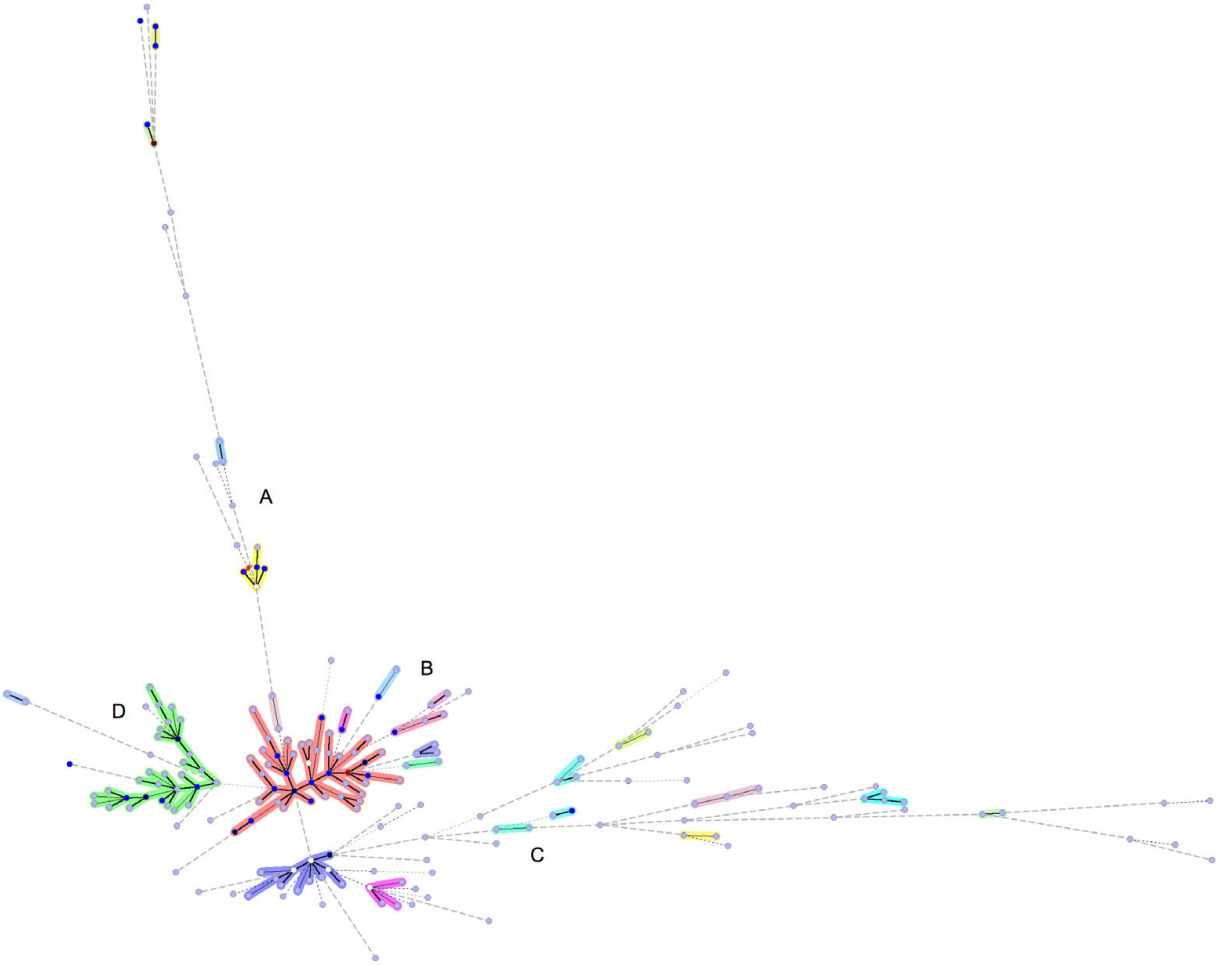
**Minimum spanning tree**. It was constructed using the 1-Jaccard index and combining numerical analysis based on both spoligotyping and VNTR results. Options were chosen on BioNumerics software to allow the construction of hypothetical genotypes (which have not been censused in French Guiana), materialised on the tree as white circles. The four major branches are called A, B, C and D. Branch D is homogeneously constituted by LAM genotypes. Branch A contains genotypes from T, Haarlem, Beijing, LAM and X families; branch B contains genotypes from Haarlem, T, S and X families; branch C shows genotypes from Haarlem, EAI, LAM, likely Haarlem and *M. africanum*.

Isolates of the T family predominated in our study (32.6% of all isolates, 31.7% of all STs). The second major clade was the LAM family (23.3% of all isolates, 27.5% of all STs) followed by the Haarlem family (22.6% of all isolates, 10% of all STs), the EAI family (12.2% of all isolates, 20% of all STs) and finally the X family (5.8% of all isolates, 6.7% of all STs). Other families (*M. africanum*, Beijing, S) were under-represented in our setting. Figure 3 illustrates the complete cluster-graph, with all the 120 different spoligotypes censused in French Guiana through 1996 to 2003. The different clades are represented with different colours, and the size of nodes is proportional to the corresponding cluster size, *i.e. *the number of isolates per cluster. The cluster-graph reveals the likely ancestral genotype of each clade. Some edges were missing to construct the complete cluster-graph and were added, unless they were not censused in French Guiana during our survey (29 spoligotypes added, for example ST 119, putative ancestor of the X superfamily). In comparison, Figure 4 represents the minimum spanning tree produced using the BioNumerics software. Combined numerical analysis of spoligotyping and VNTR data for all 344 isolates underlined at least three well-defined branches (called A, C, D) emerging from a central cluster called B. When considering each branch in detail, it can be shown that identified subgroups are most of the time not phylogenetically informative, *i.e. *isolates present in a branch are not family specific, except for branch D which is homogeneously constituted by LAM genotypes. Branch A contains genotypes from T, Haarlem, Beijing, LAM and X families; branch B shows genotypes from Haarlem, T, S and X families; and branch C contains genotypes from Haarlem, EAI, LAM, likely Haarlem and *M. africanum*.

### Estimation of transmission

Applying the indices previously described to our French Guiana data set gives the following values: RTI*n *= 0.794, RTI*n-1 *= 0.651, and TMI = 0.146, with *n *= 344 isolates, *n*_*c *_= 273 isolates in cluster, *c *= 49 clusters, *g *= 120 spoligotypes and *v*_1 _= 82 edges involving a 1-step change. The number *v*_1 _is equal to the number of genotypes *g *in the data set minus the number of connected components, which is calculated using the cluster-graph (Figure 3). The RTI values here are very high compared to the same statistic computed on spoligotypes data (TMI), which means a lower recent transmission rate evaluated with TMI method. This difference is further explained in the discussion.

## Discussion

### Molecular typing results

Our genotypic results seems to show a low level of TB transmission as evidence through: (1) a high diversity of spoligotypes (120 individual patterns for 344 isolates), (2) a low mean cluster size (Table 1; Figure 2A) in spite of the high level of clustering (79.4% of isolates included in clusters; Table 1), and (3) a high degree of unique STs (59.2%), *i.e. *ST that cannot be related to a recent event of transmission. Moreover, the biggest clusters (48 and 39 isolates respectively; see Figure 2A) concern ubiquitous spoligotypes 50 and 53, widely spread on all the five continents (spreading index SI equal to 20.95 and 28.09 respectively [30]), and for which interpretation in terms of recent transmission remains difficult. However, we should keep in mind the suboptimal choice of the spoligotyping-VNTR genotyping scheme, which provided only a slight or no improvement compared to spoligotyping only. However, for practical reasons, and given the time frame covered (1996–2003), a full MIRU12 retrospective characterization of our sample could not be performed.

### Cluster-graph and minimum spanning tree

**Figure 5:**
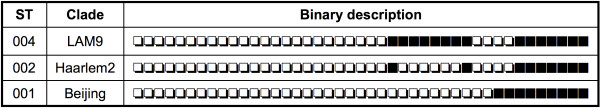
**Representative spoligotyping patterns of ST 01, 02 and 04, and their associated clades**. Dark squares indicate the presence of the spacer, and white squares indicate its absence (loss of spacers by a deletion mechanism). Unless they show some similarities (common absence of spacers 1–24) the three genotypes ST 01, 02 and 04 are from different families, *i.e. *Haarlem (absence of spacer 31 and spacers 33–36), LAM (absence of spacers 21–24 and spacers 33–36) and Beijing clades (absence of spacers 1–34) respectively. This illustrates that computation of distances on some types (for Minimum Spanning Tree construction) may be misleading.

Comparison between two clustering methods underlined major differences. The manual, parsimony-based, cluster-graph method provides more coherent results, even if, in this study, we did not consider VNTRs to build the graph. The cluster-graph construction takes into account spoligotype evolution mechanisms, *i.e. *successive loss of one to many consecutive spacers by a deletion mechanism [31] whereas the algorithm used by BioNumerics takes into account the maximal similarity between spoligotypes when considering the occurrence of the 43 spacers with respect to chronology from 1 to 43. However such similarities do not systematically correspond to existing evolutionary mechanisms. Besides, it is well known that MST provides a distance-based analysis that may not be considered as a true phylogenetic tree. One example is spoligotypes 4, 2 and 1, which were phylogenetically related to branch A of the MST (Figure 4), based on identical absence of contiguous spacers 1 to 24, as illustrated in Figure 5. In fact, those genotypes are from different families, *i.e. *Haarlem (classification rule: absence of spacer 31 and spacers 33–36), LAM (absence of spacers 21–24 and spacers 33–36) and Beijing clades (absence of spacers 1–34) respectively [32]. Finally, minimum spanning trees are more effective in case of exhaustive recruitments, in a very circumscribed and closed human community, which is not really the case in the present study.

### Phylogeography from spoligotyping

Our results are coherent with migration and colonization history of human populations in French Guiana. The Haarlem clade (23% of our strains) is considered to be a clade of European descent [33-35] and related spoligotypes might have been introduced during previous colonization of the three Guiana (*i.e. *French Guiana, Suriname and Guyana) by migrants from France, Holland and Great Britain, respectively. Occurrence of 23% of spoligotypes belonging to the LAM clade (Latin-American and Mediterranean) might be best explained by population flows with neighbouring Latin-American countries, in particular Brazil, or by historical Lusitanian or Spanish settling. Concerning the EAI clade (East-African Indian; 12% of STs), the absence of many likely-ancestral STs was noticeable (*e.g. *ST 236, the putative EAI ancestor, and 7 out of 10 absent STs on the one-mutation circle illustrated in Figure 3), whereas distant genotypes were censused, presumably indicating a more recent expansion. The genotypes present in French Guiana might have been introduced by successive arrivals of Hakka settlers from Taiwan, Singapour, Hong-Kong, Vietnam and continental China since 1820 , and more recently, by settlers from the Lao-Hmong community which arrived in French Guyana in 1977 [36]. But there are obvious limitations to what can be achieved by spoligotyping, and the dominance of T strains in the current study (33%) may (at best) demonstrate one of these. The T clade indeed remains an ill-defined ubiquist family of *M. tuberculosis *found all over the world [33,34], thus providing few phylogeographical information.

### Recent TB transmission in French Guiana

The calculated TMI indicates a recent transmission rate of 14.6% on the 1996–2003 period, instead of 79.4% and 65.1% of recent TB transmission calculated with RTI*n *and RTI*n-1 *methods respectively. In a previous study [22], the rate of recent transmission was calculated to be 49.3% in French Guiana, using the RTI*n-1 *method based on the combined spoligotyping and VNTRs genotyping. The difference between our RTI*n-1 *result and the previous study is probably due to a different sampling and a smaller time frame of the present study (396 strains from 1994 to 2003 in [22] as compared to 344 isolates from 1996 to 2003 in the present study). Comparing RTI and TMI indices, we obtained a lower ongoing transmission rate with TMI method. The three methods are based on the same hypothesis about the potential epidemiological link between patients sharing the same *M. tuberculosis *strains. But the major weakness with RTI methods is their failure to account for strains diversity and mutation. The relationship between sample size *n *and the number of observed genotypes *g *is complex and depends on sampling, mutation, and population history, which were not integrated in previous RTI indices. In addition to being useful in visualizing data, cluster-graphs provide information about the abundance of particular genotypes in the sample, as well as partial information about possible evolutionary relationships, and can then be used as a way of understanding indices measuring the severity of outbreaks. The TMI result is the only one to be coherent with genotyping results described above (high genotypic diversity, small clusters, many orphans) which supposed a low level of recent TB transmission. The concomitant hypothesis is an endemic mode of TB transmission in French Guiana, with little local contamination occurring from person to person, but an overall persistence of the disease due to the large number of imported cases from countries with high TB incidence rates.

### New "transmission mutation index" method

There are at least two biases that can occur when calculating TMI, and that may conduct to strictly opposite conclusions. The first possible bias is the emphasis of a single epidemic picture, whereas there are several parallel epidemics. For example, when considering two genotypes g1 and g2 differing by one single mutation step, the two isolates will be associated in the cluster-graph and thus related to a same epidemic, unless they potentially acquired the related genotypes independently by convergence, due to homoplasia. The infinite allele model, which underlies the TMI method, does not take into account such possibility, which can definitely occur.

The second possible bias is exactly the opposite, *i.e. *the emphasis of several epidemics, whereas there is only one. A sample is, by nature, incomplete. Thus, when isolates included in a cluster-graph are sampled from a population, some cases might be missing, which will induce a possible under-estimation of a cluster size, and/or over-estimation of the number of different clusters. If one intermediate genotype is missing between two isolates, we will detect two epidemics unless there is a single transmission event.

A third bias can also be discussed here, considering the hypothesis we made that, when person A contaminates person B, their TB strains may differ at maximum by one mutation event. In fact, this hypothesis is likely to be true for spoligotypes, which have a low mutation rate. So, the possible bias due to points 1 and 2 are believed to be greater than this last one.

We must also notice that, because genotyping requires the availability of a viable isolate of *M. tuberculosis*, the population for which genotyping is performed must be a subset of all cases with positive cultures. In this study, 342 of 345 confirmed cases were spoligotyped. In turn, cases with positive cultures must be a subset of all cases (culture positive, negative, or not done). A previous study on the Ile-de-Cayenne, French Guiana, showed that 61.7% of identified cases were confirmed by isolation and cellule culture of *M. tuberculosis *[15]. This must be taken into account when interpreting our results.

Last but not least, one should also pinpoint that no true analysis of patient demographical and medical data could be performed within the frame of this study. This is no doubt a strong limitation, since it is well known that molecular epidemiology performs well when collaboration between conventional and molecular epidemiology is achieved. The main reason relates to the difficulty to provide adequate human resources that would work for such a long period on the epidemiology of tuberculosis; however, even if a careful case-by-case analysis of the patient file data could not be assessed within the frame of the study, we fully assume that our results are a mirror of the epidemic situation of tuberculosis in French Guiana.

## Conclusion

Our results confirmed that the use of cluster-graphs from spoligotyping data to derive a TMI index provides more sensitive results than previous RTI indices in order to measure the severity of outbreaks. It allowed us to show an endemic mode of TB transmission in French Guiana associated to low local transmission of the disease, with both resurgence of old spatially restricted genotypes (endemic to French Guiana), and an importation of new TB genotypes by migration of TB infected people from high-incidence countries. Endemic genotypes might also be due, to a lower extent, to local mutation events. Those results highlight the need for specific strategies of TB control in this region, considering those new epidemiological hypotheses.

This study also suggested that our methods choice in order to get a clear picture of the TB snapshot in a high prevalence setting such as French Guiana was indeed suboptimal. Genotyping of pathogens is a fast moving field that also requires long-term experience and stability of methods to provide golden standards in molecular characterization. A decade of IS6110-RFLP predominance, has now been efficiently challenged by the power of the Multi Locus VNTR analysis (MLVA) approach, which has proven to be useful for many pathogens. Meanwhile, the spoligotyping approach, once thought to be not discriminative enough, has revealed to be an excellent long-term choice in TB genotyping, not only because it is cheap and robust, but also because the underlying role of the DR (a member of the CRISPR family of repeats) is now better understood [37], and because updated databases are regularly maintained. An ultimate player in this game, *i.e. *SNP genotyping, could become the most useful tool during the next decade [38,39]. However, for the time-being, there is no emerging consensus on what is the best genotyping scheme to understand TB transmission based on molecular characterization of clinical isolates.

## Competing interests

The author(s) declare that they have no competing interests.

## Authors' contributions

VG conceived the study, completed the data analysis and prepared the final draft of the manuscript. CS participated to the data gathering and provided expertise in phylogeographical data analysis and manuscript writing. KB participated in experimental design and molecular typing. JFG helped participate in study design. NR supervised the identification and drug-susceptibility testing of tubercle bacilli, generated the molecular genetic data and participated in data analysis. All authors read the manuscript, participated in editing the manuscript and approved the final version.

## Availability and requirements

At the time of the matching analysis, the updated spolDB4 version contained 31,642 patterns distributed into 2,393 shared types in 114 countries (an online version of this database is now available at ).

The genotypes present in French Guiana might have been introduced by successive arrivals of Hakka settlers from Taiwan, Singapour, Hong-Kong, Vietnam and continental China since 1820 , and more recently, by settlers from the Lao-Hmong community which arrived in French Guyana in 1977.

## Pre-publication history

The pre-publication history for this paper can be accessed here:


